# Assessment of depth of sedation using Bispectral Index™ monitoring in patients with severe traumatic brain injury in UK intensive care units

**DOI:** 10.1016/j.bjao.2024.100287

**Published:** 2024-05-28

**Authors:** Callum Kaye, Jonathan Rhodes, Pauline Austin, Matthew Casey, Richard Gould, James Sira, Shaun Treweek, Graeme MacLennan

**Affiliations:** 1 NHS Grampian, Aberdeen, UK; 2 University of Aberdeen, Aberdeen, UK; 3 NHS Lothian, Edinburgh, UK; 4 University of Edinburgh, Edinburgh, UK; 5 NHS Tayside, Dundee, UK; 6 Leeds Teaching Hospitals NHS Trust, Leeds, UK

**Keywords:** Bispectral Index Monitor, critical care, deep sedation, intracranial pressure, traumatic brain injury

## Abstract

**Introduction:**

Severe traumatic brain injury affects ∼4500 per year across the UK. Most patients undergo a period of sedation to prevent secondary brain injury, however the optimal sedation target is unclear. This study aimed to assess the relationship between the electroencephalogram (EEG)-based Bispectral Index™ (BIS™) value and the clinical sedation score, along with other clinical outcomes.

**Methods:**

Patients with severe traumatic brain injury in four UK ICUs were recruited to have blinded BIS data collected for a 24-h period while sedated on the ICU. Drug, physiological, and outcome data were recorded from the ICU record. Sedation management was at the discretion of the ICU clinical team.

**Results:**

Twenty-six participants were recruited to the study. The mean BIS was 38 (inter-quartile range 29**–**44) and there was poor correlation between BIS and sedation score as a group (correlation coefficient 0.17, 95% confidence interval 0.08–0.26), however the spread in BIS values increased with decreasing sedation score. There was no statistically significant relationship between BIS and intracranial pressure, vasopressor use, osmotherapy use, or need for an additional sedative.

**Conclusion:**

This study supports previous work showing that BIS decreases with decreasing sedation score. However, the variation in BIS values increased with deeper levels of clinical sedation. Patients may not be benefiting from the full potential of sedation in traumatic brain injury and further studies of sedation titrated to an EEG-based parameter are needed.

**Clinical trial registration:**

NCT03575169.

Traumatic brain injury is a common cause of physical disability in working-age adults and affects ∼4500 adults a year across the UK.^1^, ^2^, ^3^, ^4^ Despite being common, the outcomes for such patients are poor even in modern healthcare systems, with >67% of patients either dead or severely dependent after a year.^5^ In addition to the impact on patients, there is also a significant demand on health service resources, with an individual cost of excess care of >£1.36 million over a patient's lifetime.^3^ The treatment of such patients has not changed over the last decade and follows a strategy aimed at reducing the cerebral metabolic requirement for oxygen (CMRO_2_) and controlling any increase in intracranial pressure (ICP).^6^^,^^7^

Traditionally this involves a period of i.v. sedation, which is known to help achieve both these outcomes.^8^ The sedatives are titrated according to repeated neurological assessments by the bedside nurse. There is, however, increasing evidence from the non-brain injured ICU population that excessive sedation is associated with worse patient outcomes, including mortality and functional outcome.^9^^,^^10^

The most common method to assess depth of sedation in the ICU is a clinical sedation score such as the Richmond Agitation Sedation Scale (RASS).^11^ However, this has been shown in smaller studies to be a poor reflection of how sedated the brain is when assessed using an electroencephalogram (EEG), with two clinically similar patients having an ∼30% difference in underlying activity.^12^

EEG interpretation is a specialised skill and is beyond the competency of most intensive care doctors and nurses. However, because of the benefit of understanding underlying brain activity, a range of processed EEG (pEEG) monitors have been developed. These monitors analyse the raw EEG recording and convert it into a value (either numeric or categorical) which clinicians can use to guide sedative dosing. These are widely used in the operating theatre setting and have been recommended by national anaesthesia societies in the UK as part of routine monitoring for those who are anaesthetised using i.v. sedation to prevent accidental awareness under general anaesthesia,^13^ which has an incidence of ∼1:19 600 anaesthetics.^14^ However, their use in UK ICUs is low, with only seven out of 31 (23%) neurosurgical centres using BIS as part of their clinical practice.^15^ With recent international consensus statements supporting the use of continuous processed EEG monitoring, its use is likely to increase.^16^

One of the most widely used monitors is the Bispectral Index™ (BIS™). This monitor uses a single pre-formed electrode strip placed across the forehead. The monitor analyses the EEG and reports a number from 0 to 100, representing a spectrum from an isoelectric EEG (no brain activity [BIS=0]) to an alert and orientated patient (BIS=90–100).^17^ Titrating the sedation intraoperatively to maintain a BIS value of 40–60 is associated with a reduction in awareness under anaesthesia of 82% (95% confidence interval [CI] 17–98%).^18^ The monitor is easy to use, with minimal training required and may provide an alternative method of assessing sedation and guiding optimal sedative dosing in the ICU.

In this study, we aimed to assess the level of sedation of brain-injured patients in UK intensive care units using the BIS monitor, identify the relationships between BIS value and clinical outcomes, and describe the sedation practices used. This is not the first time BIS has been investigated in the setting of traumatic brain injury. Work has been done to identify the BIS score associated with optimal cerebral blood flow, when autoregulation is impaired.^19^ This concept of BIS_Opt_ is the topic of further research to prevent secondary injury caused by impaired autoregulation; however, it does not necessarily translate to preventing ischaemic-related injury.

## Methods

### Ethics

The study protocol and participant facing documentation were approved by both the Scotland A (18/SS/0100) and Bradford Leeds (21/YH/0131) research ethics committees. For patients who met the recruitment criteria, an appropriate relative was approached by a member of the research team and an informed consent form signed, after the study had been explained. If a patient regained capacity before ICU discharge, a member of the research team explained the study and took consent to remain in the study or gave the opportunity to withdraw and have their data removed.

### Study design and participants

The study initially began as a single-centre observational study in the intensive care unit at Aberdeen Royal Infirmary and then extended to three additional centres (Edinburgh Royal Infirmary, Ninewells Hospital, Leeds General Infirmary) because of slow recruitment in Aberdeen for ∼2 yr. The study was prospectively registered on ClinicalTrials.gov (NCT03575169), which was updated as the study progressed. Patients were prospectively screened and could be included within 24 h of admission to the ICU as long as they:

• Were 18 yr old or more at the time of admission
• Had a diagnosis of traumatic brain injury
• Were expected to require >24 h of sedation and mechanical lung ventilation.

This final criterion was asked of the treating clinician to ensure that patients were not recruited who were about to have sedation stopped or were not inadvertently sedated for longer than clinically indicated.

Exclusion criteria included:

1. The brain injury was considered to be unsurvivable
2. Frontal decompressive craniectomy before recruitment
3. Use of ketamine at recruitment or planned use within 24 h
4. Fractured frontal bone or severe overlying soft tissue injury
5. Simple extradural haemorrhage with no other obvious intracranial abnormality
6. Pregnancy
7. Inability to gain consent within 24 h of admission to the ICU.

Criteria 2 and 4 were as a result of the surgery/trauma site interfering with the validated site for BIS electrode placement. Criterion 3 is a result of concerns around the impact of ketamine on the calculated BIS value. Criterion 5 aimed to prevent patients with relatively minor brain injuries, who were unlikely to ever benefit from a period of sedation, from being recruited.

A target sample size of 30 participants was decided on as achievable and sufficient to provide informative BIS data. Eligible, consented participants had a Medtronic BIS™ Quatro 4XP sensor applied to their forehead as per standard instructions from the manufacturer. After confirming the electrode was functioning correctly, alarms related to BIS monitoring were deactivated to prevent clinicians altering sedative dosing based on the BIS value. The BIS EEG trace and value were hidden from the main screen. Members of the research team intermittently reviewed the monitor logs to ensure data were still being collected but did not share the results with members of the clinical team. If it was identified that the sensor was not collecting data, a fresh sensor was applied. The sensor was removed at 24 h from the time of the first electrode being applied.

### Data collection

Basic information was collected for each patient including age, gender, weight, pre-sedation Glasgow Coma Scale (GCS), and predominant injury type (i.e. diffuse axonal injury, subdural haemorrhage, contusions, mixed). For the 24 h from recruitment, hourly data were collected, including: BIS value, sedative dosing, clinical level of sedation (RASS) and use of therapies for ICP. Participants on alternative vasopressors, such as metaraminol, were converted to noradrenaline (norepinephrine) equivalents using previously published dosing equivalents.^20^

Outcome data were collected on each patient, including status at ICU discharge (alive/dead), days of mechanical ventilation, length of ICU stay, GCS at ICU discharge, and need for tracheostomy. Adverse events at the electrode site such as prolonged erythema (>1 h), blistering, or burns were recorded.

### Outcomes

The primary outcome was the mean BIS of sedated traumatic brain injury patients during the first 24 h of the ICU admission. Secondary outcomes included:

• ICP control
• Osmotherapy use
• Vasopressor use
• Sedative use
• ICU length of stay
• Duration of mechanical ventilation.

### Statistical analysis

The primary outcome was reported with descriptive statistics. Relationships between ordinal numerical variables were analysed using linear regression with a mixed-effects model. Relationships with a continuous variable and binary outcome were analysed using binary logistic regression with a mixed-effects model, with the participant being a random effect within the model to take into account repeated measures per participant. For comparing BIS values with RASS, a repeated measures of correlation test was performed. Statistical analysis was performed using R (V4.3.1, R Foundation for Statistical Computing, Vienna, Austria) with the following packages: tidyverse, lme4, and rmcorr.

## Results

A total of 26 participants were recruited, with patient characteristics and injury data presented in Table 1. Twelve participants were recruited from site 1, eight from site 2, four from site 3, and three from site 4. The majority (22, 85%) of the participants were male, which is in keeping with the epidemiology of severe traumatic brain injury.^21^ The most common injury type was mixed (eight), followed by subdural haematoma (six). The median GCS was 5.5 (4–6.04). Twenty-two participants (84.6%) survived their ICU admission.

**Table 1:** Details of participants, severity of injury, and predominant injury type. IQR, inter-quartile range.

Variable	*N*=26
Age (yr) (median [IQR])	41 (23.75–55)
Weight (kg) (median [IQR])	80 (70–80.2)
Glasgow Coma Scale (GCS) at presentation to emergency department (median [IQR])	6 (4–8)
Male, *n* (%)	22 (85)
Predominant injury	
Diffuse axonal injury, *n* (%)	4 (15)
Extradural, *n* (%)	1 (4)
Cerebral contusions, *n* (%)	6 (23)
Subdural haemorrhage, *n* (%)	6 (23)
Mixed, *n* (%)	9 (35)

### BIS values

**Fig 1:**
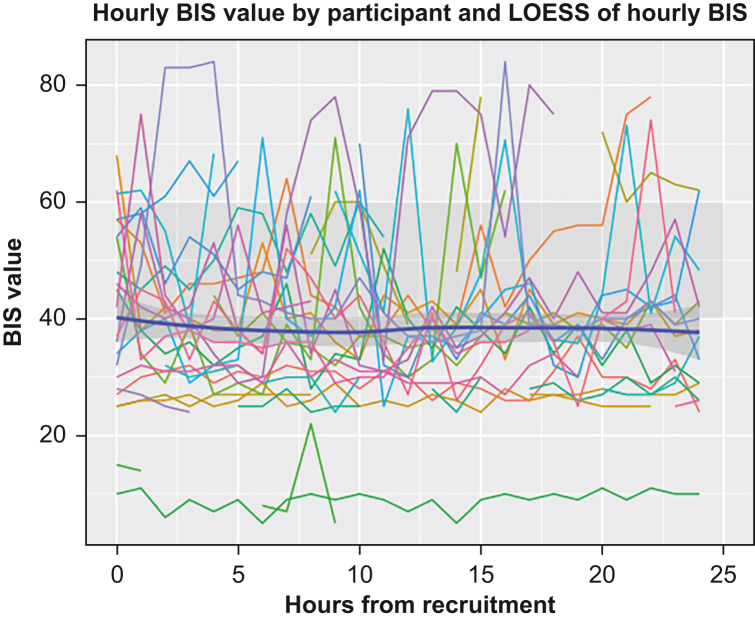
Hourly BIS value per participant (coloured lines) and smoothed line produced by locally estimated scatterplot smoothing (LOESS) of BIS for population per hour. Dark shaded area is 95% CI of LOESS; light shaded area is traditional target BIS range for general anaesthesia. BIS, Bispectral Index; CI, confidence interval.

BIS values were collected for all 26 participants. Completeness of recording was variable between participants with a mean 56% (inter-quartile range [IQR] 25**–**84%) of a potential 650 recordings available. The mean BIS value for the study population was 38 (IQR 29**–**44) with 495 individual recordings. As a group, there was no overall increase or decrease in BIS over the course of the study period (Fig. 1). One participant had a BIS <20 for the entire study period. This participant survived, with good neurological outcome at ICU discharge.

### BIS values and RASS

**Fig 2:**
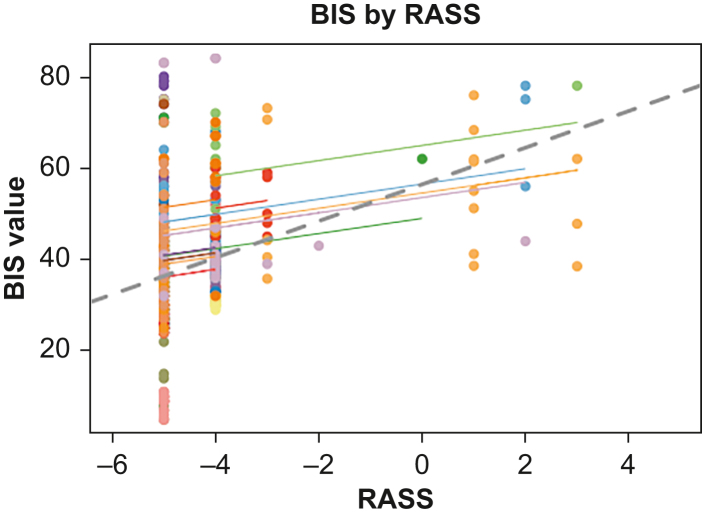
Bispectral index (BIS) variables by Richmond Agitation Sedation Scale (RASS) score. With individual repeated measures of correlation (rmcorr) line for each participant. The grey dashed line represents the overall regression line.

When comparing BIS values with the RASS score (the primary target for sedative titration in all contributing centres), there was poor correlation between the BIS and the observed RASS with BIS decreasing with decreasing RASS (*r* 0.17, 95% CI 0.08–0.26), and with the variation in BIS values increasing as the RASS decreased (Fig. 2).

### ICP control

ICP was measured during 416/650 (64%) observations, with a median ICP of 10.5 mm Hg (IQR 7–17 mm Hg). There were 29 episodes of ICP >20 mm Hg. Comparing ICP with BIS value, all but one episode of intracranial hypertension (ICP >20 mm Hg) occurred in participants with a BIS <40. The effect of BIS on ICP was statistically non-significant, with positive coefficient of 0.05 (95% CI −0.006 to 0.10, *R*^2^=0.68).

### Osmotherapy use

There were 35 episodes of osmotherapy use across seven individual participants out of 450 (7.8%) observations where a corresponding BIS value was recorded. There was a mix of hypertonic saline and mannitol. One participant was receiving a continuous infusion of hypertonic saline. There was no significant relationship between the use of osmotherapy and BIS (odds ratio 1.04, 95% CI 0.94–1.1, Akaike information criterion [AIC] 126.3, Bayesian information criterion [BIC] 138.7).

### Vasopressor requirements

The median norepinephrine dose was 0.11 μg kg^−1^ min^−1^ (IQR 0.04**–**0.23). The effect of BIS on vasopressor dosing was statistically non-significant, with a negative coefficient of −6.74×10^−4^ (95% CI −1.46×10^−3^ to 1.03×10^−4^, *R*^2^ 0.71).

### Sedation

The most commonly used (96%) primary sedative was propofol with a median dose of 1.95 mg kg^−1^ h^−1^ (IQR 1.42–2.14 mg kg^−1^ h^−1^). There was a negative correlation between BIS value and propofol dose, with a linear regression coefficient of −4.89 (95% CI −6.23 to −3.56, *R*^2^ 0.65). There was no significant relationship between BIS value and the need for an additional sedative (odds ratio 1.03, 95% CI 0.83–1.27, Akaike information criterion 68.9, Bayesian information criterion 81.5).

At participant level there were few changes to the propofol dosing each hour over the study period. The median number of changes of propofol dosing during the study was 2 (IQR 0**–**3.75); however, changes to sedative dosing did not correlate with changes in clinical sedation score (RASS).

All but two participants received a propofol and alfentanil sedative regimen. One participant received midazolam, rather than propofol, and another received remifentanil, rather than alfentanil. One participant received an additional sedative (midazolam) on top of propofol and alfentanil. Four patients received infusions of neuromuscular blocking agents at some point during the study period.

### ICU length of stay

The mean ICU length of stay was 18.2 days (IQR 7.25–21.25). There was no correlation between length of ICU stay and mean BIS per participant.

### Duration of mechanical ventilation

The mean duration of mechanical ventilation was 14.6 days (IQR 6–18.75 days). There was no significant correlation between duration of mechanical ventilation and mean BIS per participant.

## Discussion

This study confirmed that the RASS is a poor representative of underlying brain activity at a patient level. This is in keeping with other studies in the traumatic brain injury and general ICU setting which have observed similar results when comparing BIS value with RASS^12^^,^^22^ and other sedation scales.^23^ If the benefit of sedation is a reduction in the underlying brain activity, current practice may leave a significant proportion of patients sub-optimally sedated, potentially exposing them to preventable secondary brain injury and its associated morbidity.^24^ Additionally, several patients may be over-sedated leaving them exposed to the wider complications of excessive sedation such as nosocomial infection, increased ICU length of stay, and duration of mechanical ventilation, and contributing to adverse organisational factors such as increased bed occupancy and cost.^25^

An important aspect to consider in this setting is the validity of the Bispectral Index^TM^ monitor in the damaged brain. BIS involves the use of a proprietary, classified, algorithm which is applied to the EEG tracing.^17^ After initially being used as a monitoring tool, its use increased after the B-Aware trial which showed a decrease in the incidence of accidental awareness under general anaesthesia.^18^ However, many studies on the BIS monitor only recruited patients without underlying brain pathology, therefore it is unclear how the monitor interprets the damaged brain. It is possible that in this study, the low BIS values may represent a spectrum of healthy, sedated brain to damaged, unsedated brain. However, if the theoretical benefit of sedation is to reduce the activity of the brain and its requirement for oxygen, so reducing ischaemic damage, even if the monitor is showing damaged brain, it does not necessarily mean sedation will provide any additional benefit.

Further, the assumption that the use of BIS is appropriate to gauge sedation in the setting of a damaged brain could simply be erroneous. In health, the correlation—albeit with important confidence limits—between RASS and BIS is the basis of its use to reduce the risk of awareness. However, in the presence of neurological damage the situation could be much more heterogeneous. For example, as the BIS monitors regional brain activity, focussing on the frontal lobes, low values might be expected in the presence of frontal lobe injury. Paradoxically, disinhibition of the frontal lobes could be expected to manifest as increased agitation. A low BIS in the presence of an increased RASS might therefore be expected.

One of the main purported benefits of sedation in the traumatic brain injury patient is the ability to control ICP. In this study, patients who experienced episodes of intracranial hypertension and treatment for raised ICP were likely to have a lower BIS value. Raised ICP is more likely in the more severely injured brain and this study questions whether deep sedation is beneficial in preventing an increase in ICP or whether it is an inevitable consequence of that injury in that patient. Additionally, as the BIS is already low in these patients any benefit in ICP seen after additional sedation may not be attributable to increasing sedative effect on the brain, but as a result of alternative effects of the sedative, such as altering cerebral blood flow through its effect on cerebrovascular resistance.^26^^,^^27^

As previously stated, optimising sedation could be crucial to help reduce secondary brain injury, maintaining the best cellular environment for neuronal recovery. Investigating the relationship between BIS and neuronal wellbeing was beyond the scope of this study. However, future work might benefit from the incorporation of microdialysis sampling or the measurement of circulating biomarker concentrations to better understand the meaning of BIS data in patients with traumatic brain injury.

In addition to examining the relationship of BIS to other clinical variables, this study assessed the sedation practices for traumatic brain injury patients in UK ICUs. Of note was the relatively fixed dosing of sedatives, with nearly a third of participants having no change in propofol dosing throughout the study period. There are several potential reasons for this, but from our currently unpublished qualitative and survey work with ICU clinicians, it is clear that sedation dosing is not felt to be the most important aspect to consider in patients presenting with traumatic brain injury and there is a culture in ICU nursing groups that a more deeply sedated patient is a safe patient. In this context, staff judge there to be little benefit in altering dosing if the patient seems clinically safe.

### Strengths and limitations

This study has several strengths. Participants were recruited from a number of centres in a prospective manner. None of these units collaborate on the clinical care of patients and each used their own clinical guidelines for the management of sedation. Therefore, we believe the results are likely to reflect practice across the UK and in doing so reflect the underlying variation around sedation. Secondly, as the monitoring was blinded to the clinical team, the results represent the real-life sedation practices in these units and the BIS value was not used to guide clinical management. Finally, the study has shown that the BIS monitor can be used effectively in the acute stages of a traumatic brain injury in a busy ICU, which gives confidence about its potential use in further studies of BIS in this group of patients.

A number of limitations impact our findings. Firstly, although hourly BIS values were recorded, other variables from the EEG could have been recorded. These include markers of signal quality and other non-proprietary values that may have given further detail around brain activity and allowed an assessment of any electrical interference, which is common in the ICU.^28^ Additionally, although the data recorded for BIS and ICP were the recorded values on the monitor log for that hour, they may not represent the exact BIS at the time of known drug dosing or ICP at a set time. There is the possibility that transient increases in ICP may have been missed. However, as episodes of osmotherapy use were recorded, it is unlikely that there were any missed episodes of clinically significant intracranial hypertension. It is also known that a processed EEG index may be a too simplistic reflection of the EEG, and the importance of a full review of the spectrogram is required to fully interpret the EEG. However, such interpretation needs specific training and may be an unrealistic monitor of sedation for the busy ICU nurse.^29^ Finally, although the primary sedative was propofol, other sedatives were used in a small number of patients, including midazolam, which may have different effects on the BIS.

## Conclusions

This study of 26 participants with severe traumatic brain injury in four UK intensive care units showed there was no significant correlation between BIS and RASS, and there was increasing variation in observed BIS values with lower levels of the RASS scale. There was no significant relationship between BIS and ICP, vasopressor requirements, osmotherapy use and requirement for an additional sedative. With this variation in BIS for those in the clinical target range for traumatic brain injury sedation, further studies should look at the efficacy of titrating sedation to EEG-based markers such as the BIS.

## Authors’ contributions

Chief investigator and led on the development and delivery of the study: CK.

Local site leads and led on participant recruitment and data collection: JR, PA, MC, RG, JS.

Provided analysis and statistical advice and support: ST, GM.

Reviewed the submitted manuscript: all authors.

## Declarations of interest

The authors declare that they have no conflicts of interest.

## Funding

CK received a Fellowship from the NHS Research Scotland & the University of Aberdeen's Elphinstone Scholarship, which this work formed part of. JR is supported by an NHS Research Scotland Fellowship.
